# HGT: A Hierarchical GCN-Based Transformer for Multimodal Periprosthetic Joint Infection Diagnosis Using Computed Tomography Images and Text

**DOI:** 10.3390/s23135795

**Published:** 2023-06-21

**Authors:** Ruiyang Li, Fujun Yang, Xianjie Liu, Hongwei Shi

**Affiliations:** 1 College of Electronics and Information Engineering, Sichuan University, Chengdu 610041, China; liruiyang@stu.scu.edu.cn; 2 College of Computer Science, Sichuan University, Chengdu 610041, China; 2020223045216@stu.scu.edu.cn (F.Y.); liuxianjie@stu.scu.edu.cn (X.L.)

**Keywords:** prosthetic joint infection (PJI), deep learning diagnosis, multimodal fusion, CT imaging, graph convolutional neural networks (GCNs), unidirectional selective attention (USA)

## Abstract

Prosthetic joint infection (PJI) is a prevalent and severe complication characterized by high diagnostic challenges. Currently, a unified diagnostic standard incorporating both computed tomography (CT) images and numerical text data for PJI remains unestablished, owing to the substantial noise in CT images and the disparity in data volume between CT images and text data. This study introduces a diagnostic method, HGT, based on deep learning and multimodal techniques. It effectively merges features from CT scan images and patients’ numerical text data via a Unidirectional Selective Attention (USA) mechanism and a graph convolutional network (GCN)-based Feature Fusion network. We evaluated the proposed method on a custom-built multimodal PJI dataset, assessing its performance through ablation experiments and interpretability evaluations. Our method achieved an accuracy (ACC) of 91.4% and an area under the curve (AUC) of 95.9%, outperforming recent multimodal approaches by 2.9% in ACC and 2.2% in AUC, with a parameter count of only 68 M. Notably, the interpretability results highlighted our model’s strong focus and localization capabilities at lesion sites. This proposed method could provide clinicians with additional diagnostic tools to enhance accuracy and efficiency in clinical practice.

## 1. Introduction

Periprosthetic joint infection (PJI) is a catastrophic complication that may arise following joint replacement surgery. The diagnosis of prosthetic joint infections (PJI) poses a significant challenge since real evidence-based guidelines to aid clinicians in choosing the most accurate diagnostic strategy are lacking. The Musculoskeletal Infection Society (MSIS) criteria for PJI, proposed by the Second International Consensus Meeting (ICM) in 2018, offers an evidence-based definition for diagnosing hip and knee PJI [1], which presents a scoring approach to diagnosis based on the most robust evidence. However, this criteria is based on clinical performance and biochemical test results, particularly the serological, synovial, and microbiological tests, keeping its complexity during clinical use.

In fact, most signs and symptoms that might indicate the presence of a PJI may be simply related to an aseptic loosening of the prosthesis or to a soft tissue infection [2]. Radiographical examinations are widely used to detect the cause of the symptoms. Among various radiographical examinations, X-ray examination is the primary method for the evaluation of possibly infected cases, while computed tomography (CT) findings are rarely utilized as a diagnostic basis [3]. In regards to the radiological perspective, CT scans contain more invisible radiologic signs than X-ray, which achieves its significant potential value for PJI diagnosis [4]. As computer vision technology advances, deep learning techniques can be now employed to utilize imaging findings for PJI diagnosis, extracting features from invisible sites within the information-rich CT scans. Among the numerous image processing networks, transformer-based architectures exhibit exceptional texture extraction capabilities, rendering them suitable for PJI diagnosis based on CT images. Nevertheless, traditional transformer-based structures struggle to aggregate information from multiple images (generated by single CT-scan), and the immense computational cost arising from the large number of images is unacceptable. As a result, there is a pressing need for a method that can effectively aggregate information from numerous CT images.

Joint evaluation of Radiological and biochemical examination results can maximize the accuracy of PJI diagnosis; however, a unified diagnostic standard for PJI is yet to be established. As a solution, multimodal techniques can integrate imaging findings with patient text data (e.g., serological markers, medical history). There are five main challenges in the research field of multimodal machine learning. (1) Representation learning, how to represent and summarize multimodal data in a way that exploits the complementarity and redundancy of multiple modalities; (2) translation learning, to translate data from one modality to another; (3) alignment learning, to identify the direct relations between (sub)elements from two or more different modalities; (4) fusion learning, to join information from two or more modalities to perform a prediction; and (5) co-learning learning, to transfer knowledge between modalities, their representation, and their predictive models [5]. From these challenges listed above, PJI Diagnosis can be considered as a modality fusion challenge.

The deep integration of image and text modalities is the key to improving PJI diagnosis accuracy. Recent multimodel neural networks, such as ViLT [6], ViLBERT [7], IMAGEBERT [8], and UniT [9], typically manage modality fusion between text in sentences and image information. For PJI diagnosis, patient text features consist of single numerical vectors that are considerably smaller in data volume compared to images. Furthermore, due to the substantial noise in CT images, the numerical vectorized text features might show a greater correlation with PJI. Consequently, The difficulty of employing recent multimodel neural networks for PJI diagnosis arises from two main problems, (1) the overfitting of text data and (2) the excessive reliance on text data, neglecting feature extraction from images. To better utilize image data, as well as mitigate the network’s bias towards text data, it is crucial to minimize the impact of extensive noise within CT images and prevent the overfitting of text data.

**Figure 1:**
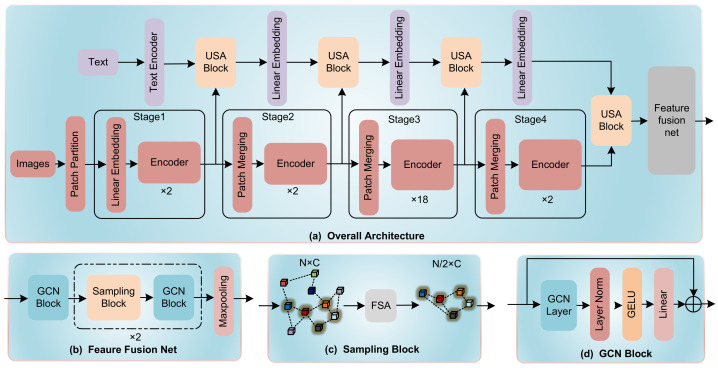
(**a**) The overall architecture of HGT. (**b**) The architecture of GCN Feature Fusion Net. The Encoder in our architecture is a ViT-based encoder. (**c**) The Sampling Block using FSA algorithm to sample from multiple features. (**d**) A GCN Block that maintains the dimensionality of input features and output features.

To address these challenges, we proposed a novel network structure, named as HGT in this study, which is a 5-stage modality fusion network based on Transformer Architecture. Three main innovations of the architecture can be elaborated in three main aspects, (1) before all stages, an image sampling strategy is proposed to reduce computational complexity by sampling the CT images derived from single-scan before the whole modality fusion process; (2) in the first to fourth stage of HGT, a Unidirectional Selective Attention (USA) is used for deep fusion between image features and text feature; and (3) at the final stage of the network, a Feature Fusion network based on graph convolutional neural networks (GCNs) [10] is proposed to fuse features between different CT images. In more detail, the Unidirectional Selective Attention (USA) enables text data to selectively attend to important features within high-noise CT image data for modality fusion. The proposed Feature Fusion network integrates features by reducing the graph complexity through a coarsening process. For this process, we develop a feature selective algorithm to sample lesion site features among all input CT images. By evaluating on self-created PJI dataset, HGT achieves state-of-the-art performance. The network structure is shown in Figure 1.

This network effectively utilizes both the numerical text data of patients and CT image features for PJI diagnosis. It substantially enhances clinical diagnostic efficiency and reduces PJI misdiagnosis rates, which has significant clinical implications for the prevention and treatment of secondary occurrence of PJI.

## 2. Related Work

### 2.1. PJI Diagnosis

Periprosthetic joint infection (PJI) diagnosis has been a topic of interest in recent years due to the increased prevalence of joint replacement surgeries. Various studies have focused on the development of new markers or the combination of existing markers to improve the specificity and sensitivity of PJI diagnosis [1,11,12]. Other research has investigated the use of imaging techniques, such as ultrasound [13], MRI [14], and nuclear imaging [15], to provide additional information on the infection status within the joint.

As a highly robust method, machine learning-based methods are capable of identifying personalized important features missing from criteria-based methods and providing interpretable decision support for individual diagnosis [16]. Klemt et al. have compared three different machine learning methods, Artificial Neural Network (ANN), Random Forest, and Elastic-net Penalized Logistic Regression in PJI diagnosis [17]. Tao et al. use the Convolutional Neural Network (CNN) to analyze the pathological sections of PJI patients under high magnification fields [18]. For greater accuracy, integrating information from different modalities (clinical performance, biochemical test results, and radiographical examinations) is highly important as imaging findings can potentially improve diagnostic accuracy. However, such diagnostic methods based on the fusion of multimodal information is still to be established.

### 2.2. Transformer

**Figure 2:**
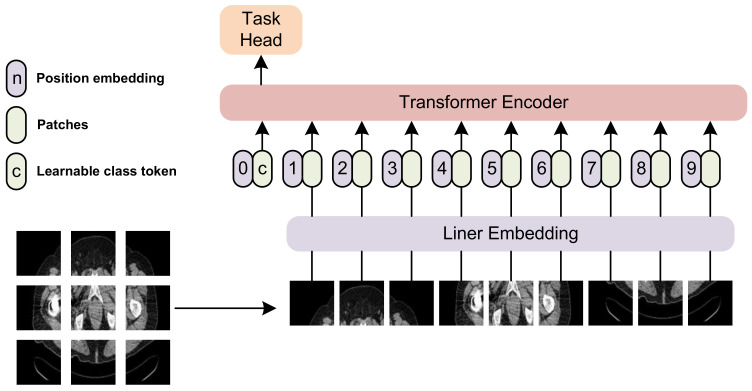
The general architecture of a ViT-based transformer. We split an image into multiple patches, and feed them into the transformer encoder. A task head is incorporated at the end of the architecture.

The transformer architecture, first introduced by Vaswani et al. [19], has become a cornerstone in the development of deep learning models for natural language processing and computer vision tasks. Transformer-based models have shown exceptional performance in a wide range of applications, including machine translation [19], text summarization [20], and image classification [21]. Specifically, ViT [21], SWIN [22], and Max-ViT [23] are transformer-based visual architectures that have shown great promise in feature extraction from images. The general architecture of a ViT-based transformer is shown in Figure 2.

In disease diagnosis, transformer-based methods have shown their high practicality. Lei et al. developed an automated diagnosis framework for COVID-19 in chest CTs based on the SWIN Transformer [24]. Jafari et al., use Deep Transformers and Explainable Artificial Intelligence for the Automatic Diagnosis of Myocarditis Disease in Cardiac MRI Modality [25]. Xu et al., have developed a hierarchical transformer for eye diseases diagnosis [26]. Nogales et al., have applied BERT in Parkinson’s Disease diagnosis. In recent trends, Transformer-based multimodal architectures are widely used for high accurate diagnosis. Xing et al., proposed a method for Alzheimer disease diagnosis by fusing multimodal visual features using a transformer [27]. Dai et al. have developed a multimodal method for image classification using a transformer [28]. Cai et al. have developed a method for skin disease by fusing images and metadata using a transformer [29]. As above, for PJI diagnosis, transformer-based multimodal architectures can be a suitable candidates for improving diagnostic accuracy.

### 2.3. GCN

Graph Convolutional Networks (GCNs) [10] are effective tools for analyzing graph-structured data. They have demonstrated success in diverse domains, including social network analysis [30], drug discovery [31], computer vision [32], and recommendation systems [33]. GCNs excel in handling irregular data structures and capturing the relational information between data points, making them an ideal choice for feature fusion.

Numerous applications exist for feature fusion based on Graph Convolutional Networks (GCNs). In the semantic segmentation field, GCNs are used as a feature fusion tool during the semantic segmentation process [34]. Apart from that, GCNs have also been used for feature fusion during hyperspectral image classification [35,36]. For traffic prediction, GCNs are used to build traffic network flow for deep feature fusion [37]. As in medical feature integration, GCNs have also been widely used. The feature derived from different medical characteristics can be easily integrated to diagnose a variety of diseases [38,39,40]. Thus, the potential of GCNs in integrating large scale radiographical features can be easily shown.

In our work, we represent a CT images graph and define GCN on it. By a proposed feature sampling algorithm (FSA), our novel GCN-based feature fusion network can easily achieve feature integration between numerous CT images with low parameters.

## 3. Methods

In our design, the proposed architecture processes multiple CT images and medical numerical vectors within a single feedforward calculation. All images belonging to a single CT scan initially undergo a sampling strategy to reduce computational complexity. Subsequently, the sampled images and medical numerical vectors are fed into the network for in-depth feature extraction and combination. Finally, the deeply fused features extracted by the first four stages of the network are fed into a GCN feature fusion network to obtain the final fused feature, denoted as $f_{out}$. This feature is directly used for diagnosing periprosthetic joint infection (PJI).

### 3.1. Sampling Strategy

For two primary reasons, directly feeding all images from a single CT scan into our proposed architecture poses challenges; (1) a single CT scan generates hundreds of images, which could significantly increase computational complexity and training difficulty; (2) the number of images produced by a single CT scan varies, leading to an uneven distribution of images within the dataset.

To effectively address these issues, a sampling strategy is applied to each CT scan. An ideal sampling strategy should select an equal number of images from different CT scans while preserving the overall features of the original CT scans. A uniform sampling strategy maintains the number of sampled images but reduces the number of used images. Alongside feature reduction, this approach may substantially decrease the network’s performance.

**Figure 3:**
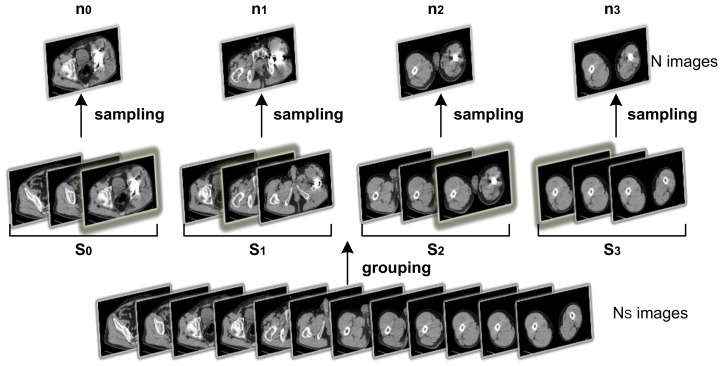
The process of sampling strategy, with N=4, $N_{S}=12$ here for illustration.

As a solution, a random distribution can be introduced on top of the uniform sampling method. Assuming the number of images generated by a single CT scan is *N*, with picture index $index_{k},k\in 0,1,\ldots ,N-1$. The target number of sampled images is $N_{S}$. The images are divided into $N_{S}$ non-overlapping groups, $S_{i},i\in 0,1,\ldots ,N_{S}-1$, based on their index. During sampling, an image within group $S_{i}$ is randomly chosen as the group’s sampled image, denoted as $n_{i}$. The final image set can be represented as $n_{0},n_{1},\ldots ,n_{N_{S}-1}$, as illustrated in Figure 3.

This method not only addresses the aforementioned issues but also allows all images in the original CT scan to be utilized by the network during training, as the sampled image sets differ each time.

### 3.2. Unidirectional Selective Attention

In mainstream transformer-based multimodal architectures, cross-attention is widely employed for feature integration between vision and text modalities. The information flow between these two modalities in this attention mechanism is bidirectional. For PJI diagnosis, the vision modality consists of high-noise CT images, while patient text features comprise single numerical vectors that are considerably smaller in data volume compared to images and could exhibit a stronger correlation with PJI. Consequently, the difficulty of employing bidirectional cross-attention for cross-modal feature integration is apparent due to the overfitting of numerical indicators data and neglect of image feature extraction. In contrast, the Unidirectional Selective Attention (USA) we propose can easily circumvent this issue.

In our Unidirectional Selective Attention (USA) mechanism, only a one-way information flow exists from the vision to numerical indicators modality. This mechanism allows the numerical indicators to directly select key information from high-noise images for modality combination, accelerating the feature fusion between image and numerical indicators information. Initially, numerical indicators features are extracted by encoding the original numerical indicators information $T\in R^{dimT}$ through a linear transformation encoder. The resulting numerical indicators feature, denoted as $F^{T}\in R^{dimT}$, can be represented as

(1) $$F^{T}=Encoder\left(T;\theta_{Encoder}\right)$$

where encoder is a linear transformation with an output dimension of dimT. Subsequently, the input image feature $F^{I}\in R^{N\times dim}$ is considered, along with the *Q*, *K*, and *V* vectors of the attention mechanism. The *Q*, *K*, and *V* vectors can be expressed as:

(2) $$Q=\Psi_{1}\left(F^{T};\theta_{\Psi_{1}}\right)$$

(3) $$K=\Psi_{2}\left(\left[F^{T},F_{0}^{I},F_{1}^{I},\ldots ,F_{N}^{I}\right];\theta_{\Psi_{2}}\right)\;V=\Psi_{3}\left(\left[F^{T},F_{0}^{I},F_{1}^{I},\ldots ,F_{N}^{I}\right];\theta_{\Psi_{3}}\right)$$

where [·] denotes the concatenation operation, and Ψ represents a linear transformation with parameters $\theta_{\Psi }$. For practical purposes, a multi-head attention mechanism is employed. Thus, the Unidirectional Selective Attention (USA) can be represented as

(4) $$USA\left(Q,K,V\right)=\Psi_{0}\left[head_{1},\ldots ,head_{h}\right]$$

(5) $$head_{i}=Attention\left(\Psi_{i}^{Q}\left(Q\right),\Psi_{i}^{K}\left(K\right),\Psi_{i}^{V}\left(V\right)\right)$$

where the attention is the same as in ViT [21], and [·] denotes the concatenation operation.

**Figure 4:**
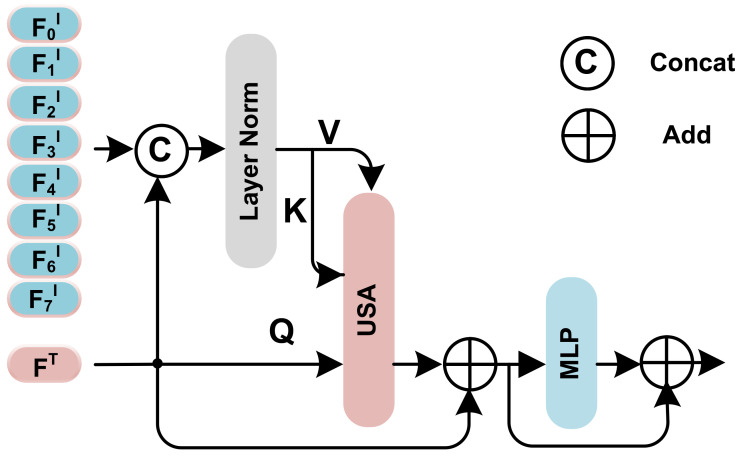
The detailed architecture of the Unidirectional Selective Attention Block. MLP here is a 2 layer Multilayer Perception with GeLU activate function [41], USA is the Unidirectional Selective Attention mechanism proposed above.

Through this mechanism, the low-noise information in the images can be selected by the numerical indicators information for more efficient modality combination. In practice, the Unidirectional Selective Attention (USA) has been incorporated by utilizing the structure depicted in Figure 4.

### 3.3. GCN Feature Fusion Net

**Figure 5:**
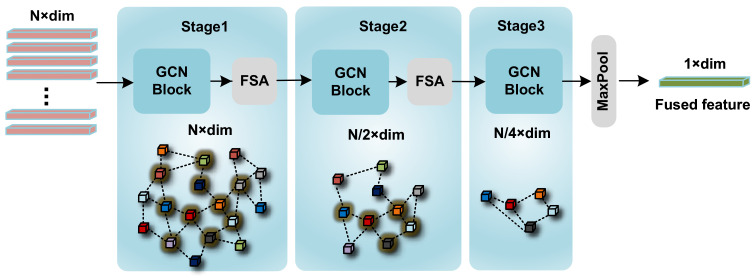
The detailed architecture of the GCN Feature Fusion Net. This intricate architecture encompasses three stages. Within each stage, the number of features progressively diminishes, with the feature count reducing by half following every FSA implementation.

After the first four stages of the entire architecture, the integrated features of numerical indicators and images can be obtained, which can be denoted as $F^{I}\in R^{N\times dim}$. To further acquire a lower-dimensional feature representation, $F^{I}$ is processed by the GCN Feature Fusion Net, as illustrated in Figure 5.

### 3.4. Graph Based on Features of CT Images and Numerical Indicators

Upon obtaining the integrated features $F^{I}$, an undirected graph *G* with *N* nodes based on the vectors $F^{I}$ can be constructed to represent the intrinsic relationships between multiple features. The adjacency matrix of this graph, $S\in R^{N\times N}$, is defined as

(6) $$S_{ij}=\Omega \left(g_{ij};\theta_{\Omega }\right)$$

where $gij=[F_{i}^{I},F_{j}^{I},|F_{i}^{I}-F_{j}^{I}{|}^{2}]\in R^{2\times dim+1}$ represents the spatial relation of two distinct image features, and [] denotes vectorized concatenation of elements. Ω is a three-layer MLP with GeLU activation function, and its parameters are denoted by $\theta_{\Omega }$, with the MLP outputting a scalar $S_{ij}$.

Next, the KNN algorithm is utilized, based on the index of the input images, to calculate the k nearest neighbors for each feature, retaining only the edges between neighboring features in *G*. Consequently, the sparse adjacency matrix *A* for *G* is obtained, defined as

(7) $$A_{ij}=S_{ij}\cdot I\left\{F_{j}^{I}\in N\left(F_{i}^{I}\right)\right\}$$

where I(·) is a binary function indicating whether $F_{j}^{I}$ is the neighbor of $F_{i}^{I}$. Clearly, the undirected graph G effectively represents the abstract relationships between the features of each image, encompassing the complete image features and numerical indicators features.

### 3.5. Image Feature Fusion and Sampling

The Graph Convolutional Neural Network (GCN) is well-suited for learning abstract relationships between nodes in an undirected graph and can learn the abstract representation of the entire graph. Let $G^{l}$ represent the feature undirected graph at the l-th stage; a forward pass of the l-th stage GCN Block in our network can be defined as

(8) $$F_{middle}^{l}=\Gamma \left(A^{l}LN\left(F^{l}\right),\theta_{\Gamma }\right)W^{l}+F^{l}$$

where $A^{l}$ represents the sparse adjacency matrix of $G^{l}$, LN represents Layer Normalization [42], $W^{l}\in R^{d\times d}$ is the learnable weight matrix, and Γ(·) is a non-linear transform comprising a Layer Normalization followed by a GeLU activation. The dimension of $F^{I}$ remains the same before and after the GCN Blocks.

Next, in order to extract global features, the undirected graph *G* must be coarsened. Let the coarsened features be defined as $F^{l+1}$. Ideally, if the feature information remains unchanged before and after coarsening, the prediction results using the pre-coarsened features $F_{middle}^{l}$ and the post-coarsened features $F^{l+1}$ should be the same. As for PJI diagnosis, if any of the features $F_{i}^{l}$ among $F^{l}$ shows positive, the diagnosis should be considered positive. The final diagnostic judgment largely depends on the most significant features among the many features in feature matrix *F*; therefore, it is necessary to retain these features in the coarsened feature matrix $F^{l+1}$ from $F_{middle}^{l}$. To meet the above requirements, a novel selective feature sampling strategy (FSA) based on the max function has been designed. The FSA strategy can be defined as:

(9) $$j=\underset{i\leq N}{\arg\max},max(\Phi \left(F_{middle,i}^{l},\theta \Phi \right)),M$$

(10) $$F^{l+1}=F_{middle,j}^{l}$$

where *N* denotes the quantity of image features prior to sampling, and *M* signifies the number of image features after sampling has occurred. The term max refers to the process of obtaining the highest value within a vector, and argmax(·) represents the extraction of index values corresponding to the top *M* largest elements present in the input vector. Φ is a two-layer MLP incorporating the GeLU activation function, and its parameters $\theta_{\Phi }$ can be effectively trained utilizing the loss described in Section 4.2, Network Training.

With this strategy, a coarsened graph $G^{l+1}$ along with its new nodes $F^{l+1}$ can be obtained. By stacking GCN Blocks and FSA, the architecture of GCN feature fusion network can be achieved. After passing the features $F^{1}$ through the GCN feature fusion network, the low-dimensional representation of fused feature can be obtained.

## 4. Implementation and Results

### 4.1. Datasets

In this study, a custom-built periprosthetic joint infection (PJI) dataset is employed. The dataset includes 103,049 black and white CT image samples and 489 numerical text samples. Each numerical indicators sample comprises 14 numerical indicators, which represent various medical and demographic factors associated with PJI. The SOMATOM Definition AS+ CT machine are utilized to generate the images, primarily employing original axial images under the CT_SOM5 SPI protocol.

**Figure 6:**
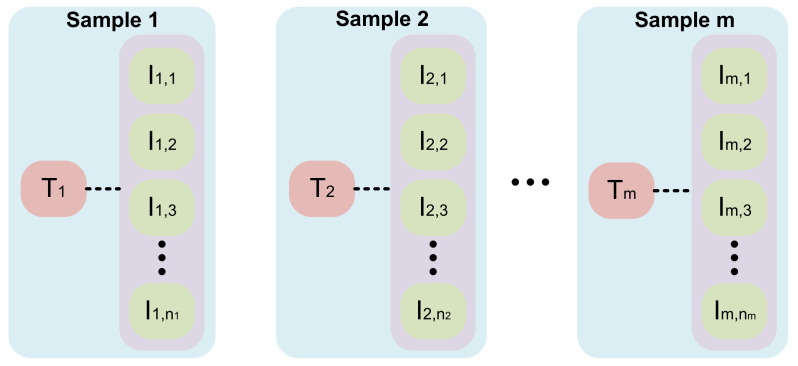
The structure of the dataset. $T_{1},T_{2},T_{m}$ represent different numerical indicators data. $I_{m,n}$ represents the *n*th image associated with numerical indicators $T_{m}$. The range of *n* varies from 150 to 400.

All numerical indicators are normalized to [0, 1]. The composition of PJI dataset is presented in Table 1, while the composition of a single numerical text sample and the relationships between PJI with 14 numerical indicators are introduced in Table 2. One numerical text sample on the dataset is associated with multiple images. One sample in our dataset contains a text sample and its related images. The structure of the dataset is shown in Figure 6.

### 4.2. Training Loss and Implementation Details

Within the comprehensive training framework, the overall loss function encompasses both diagnosis and select loss components. The total loss can be expressed as follows:

(11) $$L=L_{Diagnosis}\left(A\left(f_{out}\right),y\right)+\sum_{l=0}^{L-1}\sum_{i\in j}L_{Select}\left(\Phi \left(F_{middle,j}^{l},\theta_{\Phi }\right),y\right)$$

In this equation, *y* denotes the true label of the diagnosis, while both $L_{Diagnosis}$ and $L_{Select}$ represent cross-entropy loss. A signifies a fully connected (FC) layers with output dimensions of 2.

The model is trained for 200 epochs using a batch size of 128 (64 images per text and 2 texts in total) on a single NVIDIA RTX 4090 GPU. The AdamW optimizer is employed [43], featuring a weight decay of 0.01. The learning rate is warmed up to $1\times 10^{-4}$ in the first 5 epochs and decays to $1\times 10^{-6}$ following a cosine schedule. Images are resized to a resolution of 224×224, with augmentations such as RandomHorizontalFlip and RandomRotation applied. The numerical text indicators vector size is set to 1×14. The k-value for the KNN algorithm in each GCN layer is set to 2, while the M-values for all sampling block structures are equal to N/2. To expedite the training process, the NVIDIA AMP Strategy is utilized.

### 4.3. Evaluation Metrics

The accuracy (ACC) is a widely used performance metric that quantifies the proportion of correct predictions made by a model relative to the total number of predictions. Mathematically, it can be expressed as

(12) $$ACC=\frac{TP+TN}{TP+TN+FP+FN}$$

where TP is the number of true positives, TN is the number of true negatives, FP is the number of false positives, and FN is the number of false negatives. The area under the curve (AUC) is a performance metric that evaluates the trade-off between the true positive rate (sensitivity or recall) and the false positive rate (1-specificity) at various threshold settings. Specifically, it refers to the area under the receiver operating characteristic (ROC) curve. The true positive rate (TPR) and false positive rate (FPR) can be defined as follows:

(13) $$TPR=\frac{TP}{TP+FN}$$

(14) $$FPR=\frac{FP}{FP+TN}$$

Throughput, measured in images per second (images/s), is a performance metric commonly used to evaluate the efficiency of a machine learning model, particularly in the context of image processing and computer vision tasks. In our study, the throughput of the model can be calculated through the following equation

(15) $$Throughput=\frac{N_{I}}{T_{B}\times B}$$

where $N_{I}$ is the total number of processed images, $T_{B}$ is the total processing time of one batch in seconds, and *B* represents Batchsize

### 4.4. Experiment on PJI Dataset

**Figure 7:**
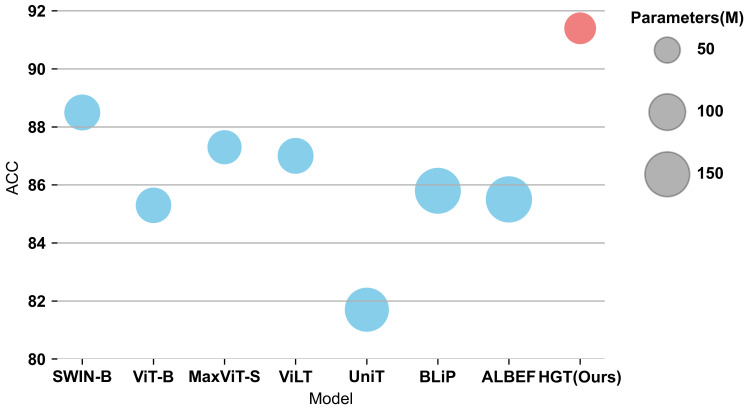
The ACC of different models under the same experiment configuration. Each circle represents a model. The size of the circle reflects the Params. of the Model.

To evaluate the model’s performance, experiments were conducted on the PJI dataset using various multimodal methods. The performance comparisons, based on the aforementioned settings, are presented in Table 3. The comparison of ACC between different models under the same experiment configuration is shown in Figure 7.

Under the basic settings, the HGT model significantly outperforms the most recent robust models. With only 68 M parameters, HGT achieves a top-1 accuracy of 91.4% and an AUC of 0.959, surpassing the most powerful model by 2.9% in top-1 accuracy and 2.2% in AUC, while utilizing fewer parameters. In summary, the proposed model outperforms other models employing MLP, merged-attention, and co-attention as their multimodal fusion methods. The substantial improvement of our proposed model demonstrates the considerable advantage of using the USA and GCN fusion network as multimodal fusion methods for PJI diagnosis.

### 4.5. Ablation Study

To assess the effectiveness of the USA Block and GCN feature fusion network, an ablation study was conducted. The results are presented in Table 4. As illustrated, the USA Block methods considerably outperform the Non-USA Block methods, yielding an increase of up to 3.5% in top-1 accuracy and 5.6% in AUC. Furthermore, the GCN feature fusion network methods exhibit significant improvement over the non-fusion network methods, with gains of up to 2.4% in top-1 accuracy and 3.6% in AUC.

**Figure 8:**
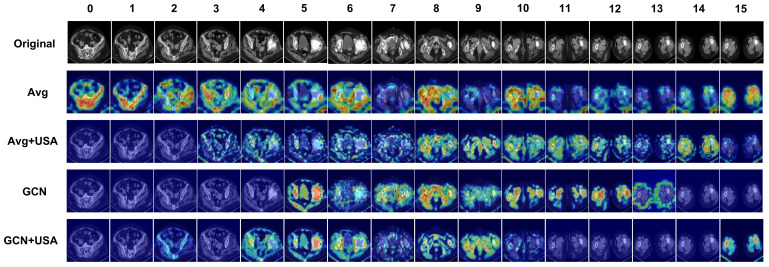
The visualization results of the methods based on ablation study settings are presented. In the Figure, “Avg” represents the Averagepool, USA represents the Unidirectional Selective Attention, while “GCN” denotes the GCN Feature FuseNet. The images with index ranging from 4 to 8 are observed to contain features indicative of infection occurrence, with the infection manifesting in the patient’s right hip bone. The images with index ranging from 0 to 3 and 9 to 15 are observed to contain no features indicative of infection occurrence.

In order to examine the implications of each block, Gradient Class Activation Mapping++ (Grad-CAM++) [46] was employed on a single PJI-positive CT scan to visualize the features extracted by different models. Overall, 16 uniformly sampled images from the same CT examination were input into the network, with the results displayed in Figure 8. It is evident that the GCN feature fusion network enhances performance by enabling a heightened focus on images related to the lesion site. Additionally, incorporating the USA Block facilitates more precise identification of regions within images where the infection occurs.

#### 4.5.1. Influence of k in Undirected Graph Construction

The value of k, an important hyperparameter in the KNN algorithm for GCN undirected graph construction, is tested for various values to assess model performance. The feature maps are visualized using Grad-CAM++ to evaluate the feature extraction capabilities.

Model performance results are presented in Table 5. It is evident that performance declines as the value of k increases. The k = 2 model outperforms the k = 4 model by 0.8% in ACC and 1.8% in AUC. The k = 4 model shows a 1.2% higher ACC and a 1.7% higher AUC compared to the k = 6 model.

**Figure 9:**
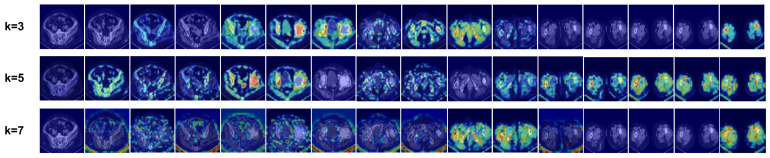
Visualization results of methods based on different k values. The original images are the same as in Figure 8.

To explain these results, the visualization outcomes are displayed in Figure 9. The ability to locate lesion sites in all CT images and focus on a single lesion within an image diminishes with increasing k values. As k increases, the GCN can aggregate more non-neighborhood features, resulting in a dilution of local features and, consequently, reduced model performance.

#### 4.5.2. Impact of Sampling Strategy

**Figure 10:**
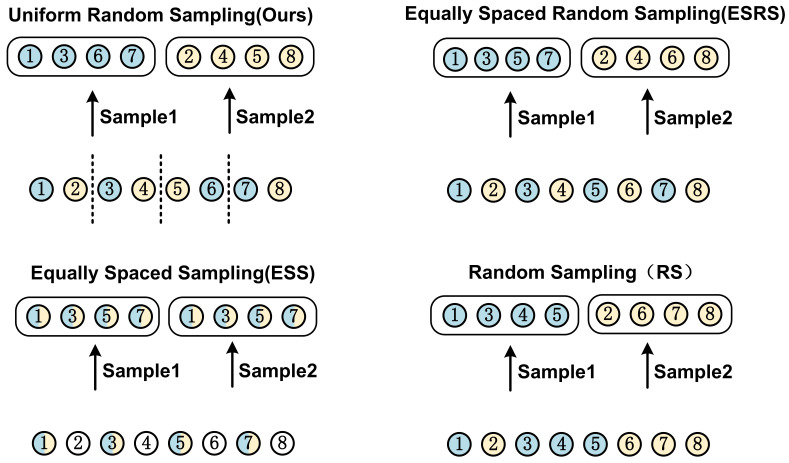
This figure illustrates the detailed mechanisms of four different sampling strategies, where two batches of images are obtained from the same CT scan using four distinct approaches.

The choice of sampling strategies can significantly impact a model’s performance. Various sampling strategies are evaluated using the same settings as the experiment on the PJI dataset, as depicted in Figure 10. The feature extraction capabilities of these strategies are assessed by visualizing the feature maps using Grad-CAM++.

The model performance results are displayed in Table 6. As observed, our sampling strategy outperforms other strategies. Our sampling strategy bring a promotion in both ACC and AUC by 2.9% and 3.6%, respectively, when compared to the least effective sampling strategy. The suboptimal performance of the equally spaced sampling strategy can be ascribed to the diminished number of CT image samples. The equally spaced random sampling strategy surpasses the equally spaced sampling strategy by 2.4% in ACC and 0.5% in AUC, which can be attributed to the comprehensive utilization of the CT image dataset. The random sampling strategy results in a performance decline due to the increased difficulty for the GCN feature fusion network in learning the topological structures of the undirected graph. The shortcomings of other sampling strategies further underscore the efficacy of our sampling strategy.

**Figure 11:**
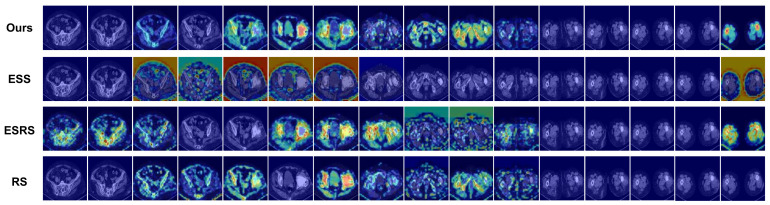
Visualization results of methods based on different sampling strategies. The original images are the same as in Figure 8.

The aforementioned analysis is corroborated by the visualization results in Figure 11. Among all strategies, our approach attains the highest concentration on lesion sites.

### 4.6. Performance on Single Modality

To assess the performance of our model within a single modality, we employed a method where data from one modality were isolated by setting the values of the other modality to zero. The results are presented in Table 7.

Observations indicate that UGT(Text) incurs a minor performance decline when compared to MLP. In contrast, UGT(Image) demonstrates a substantial improvement over SwinS, attributable to its Unidirectional Selective Attention (USA) and GCN feature fusion block. This supports the notion that our method can adeptly manage the high-noise image modality independently, even without the text modality.

## 5. Limitations

There are still limitations in our research that we hope to address in the future. For instance, the proposed method requires both CT images and numerical text data, which may not be available in all clinical settings. Although this study significantly enhances the precision and practicality of infection diagnosis using deep learning methods, it was only validated in a single self-built dataset. Therefore, further research may be needed to validate the effectiveness of the proposed method in different settings and populations.

## 6. Conclusions

In conclusion, this study introduces a hierarchical CGN-based transformer (HGT) for multimodal PJI diagnosis, utilizing CT images and patients’ numerical text data to enhance diagnostic accuracy. A distinctive sampling strategy was implemented, along with a GCN-based feature fusion approach for efficient image processing and precise infection localization. Moreover, an Unidirectional Selective Attention (USA) Block was employed to balance learning between easily acquired low-noise numerical indicators and challenging high-noise image modalities.

The proposed method was validated using a custom-built PJI dataset and compared to multiple competitive multimodal methods under the same experimental configuration. The results demonstrate that the ACC of this method reaches 91.4%, which is 2.9% higher than the most recent powerful model, and the AUC attains 95.9%, signifying a substantial improvement of 2.2% compared to other methods with fewer parameters.

In the future, we aim to expand this approach to other complications, harnessing the power of large-scale language generation models to achieve automatic diagnosis and case writing for multiple concurrent conditions.

## Figures and Tables

**Table 1:** The composition of the custom-built PJI dataset.

Modality	Positive Samples			Negative Samples			Total Samples
	**Train**	**Test**	**Valid**	**Train**	**Test**	**Valid**	
image	25,303	5517	13,222	42,106	5365	11,536	103,049
text	98	20	50	168	20	50	489
	**Total Positive Samples**			**Total Negative Samples**			
image	44,042			59,007			
text	221			268			

**Table 2:** The composition of a single numerical text sample.

Data Type	Value Range	Relationship with PJI
C-reactive Protein (CRP)	[0,120]	Serology indicators related with PJI
Erythrocyte Sedimentation Rate (ESR)	[0,120]	
Lesion Site	0 or 1	Site of prosthetic join
Sex	0 or 1	Basic information about patient
Age	[0,100]	
Hypertension	0 or 1	Diseases history may related with PJI
Diabetes	0 or 1	
Rheumatoid Arthritis	0 or 1	
Anemia	0 or 1	
Osteoporosis	0 or 1	
Cerebral Infarction	0 or 1	
Hypoalbuminemia	0 or 1	
Hypothyroidism	0 or 1	
Liver Disease	0 or 1	

**Table 3:** Performance comparison under PJI dataset settings. Throughput is measured on a single RTX4090 GPU with batch size 128. In vision modality networks, numerical text indicators information obtained from the text indicators encoder is processed through a three-layer multi-layer perception (MLP) featuring a GELU activation function, while the images are passed through the entire network accompanied by a Maxpool operation in the batch dimension. The fused image and text indicators features are directly concatenated to perform PJI prediction.

Model	Eval Size	Multimodal Fusion	Parameters	Throughput (Image/s)	ACC	AUC
SWIN-B [22]	$224^{2}$	MLP	88 M	483	88.5	92.6
ViT-B [21]	$224^{2}$		86 M	630	85.3	91.5
MaxViT-S [23]	$224^{2}$		79 M	428	87.3	93.7
ViLT [6]	$224^{2}$	Merged-attn.	88 M	624	87.0	93.7
UniT [9]	$224^{2}$	Cross-attn.	135 M	556	81.7	91.2
BLiP [44]	$224^{2}$		147 M	549	85.8	93.1
ALBEF [45]	$224^{2}$		149 M	547	85.5	93.2
**HGT**	$224^{2}$	USA+GCN	**68 M**	552	**91.4**	**95.9**

**Table 4:** The ablation study results are presented. In the Non-USA Block methods, numerical indicators information acquired from the text indicators encoder undergoes processing through a three-layer multilayer perceptron (MLP) with a GELU activation function, while images pass through the entire SWIN-S network, followed by a Maxpool operation in the batch dimension. The fused image and numerical indicators features are directly concatenated for PJI prediction. In the non-fusion network methods, the GCN feature fusion network is substituted with an Averagepool operation.

Feature Fusion Method	ACC	AUC
Avg	87.9	90.3
GCN	90.3	93.9
USA+Avg	90.0	92.9
**USA+GCN**	**91.4**	**95.9**

**Table 5:** Performance with different k values.

k	Feature Fusion Method	ACC	AUC
**2**	USA + GCN	**91.4**	**95.9**
4	USA + GCN	90.6	94.1
6	USA + GCN	89.4	92.4

**Table 6:** Performance with different sampling strategies.

Sample Strategy	Uniform Random Sampling (Ours)	Equally Spaced Random Sampling (ESRS)	Equally Spaced Sampling (ESS)	Random Sampling (RS)
ACC	**91.4**	90.9	88.5	89.4
AUC	**95.9**	92.8	92.3	93.4

**Table 7:** Performance on single Modality. HGT(Text) and HGT(Image) represents padding Image and Text indicators modalities to zero in HGT separately. MLP here is a five-layer multilayer perception with a hidden dim of [96, 192, 384, 768] and GELU activation function. In SwinS, the images are passed through the entire network accompanied by a Maxpool operation in the batch dimension.

Method Type	Text Only		Image Only	
Methods	UGT (Text)	MLP	UGT (Image)	SwinS
ACC	87.0	87.3	83.5	81.1
AUC	92.6	93.5	88.2	85.9

## Data Availability

Due to privacy restrictions, the dataset has not been published yet. You can contact the corresponding author to obtain.
